# The Use of Anabolic Steroids by Bodybuilders in the State of Sergipe, Brazil

**DOI:** 10.3390/ejihpe14050096

**Published:** 2024-05-16

**Authors:** Josué Cruz dos Santos, Erivaldo de Souza, Daniela Meneses-Santos, Carla Roberta de Oliveira Carvalho, Jymmys Lopes dos Santos, Felipe J. Aidar, Anderson Carlos Marçal

**Affiliations:** 1 Programa de Pós-Graduação em Educação Física, Universidade Federal de Sergipe, São Cristóvão 49100-000, Brazil; josue.cruz.santos@gmail.com (J.C.d.S.); proferisouza@gmail.com (E.d.S.); jymmys.lopes@gmail.com (J.L.d.S.); 2 Departamento de Morfologia, Universidade Federal de Sergipe, São Cristóvão 49100-000, Brazil; danyymeneses@yahoo.com.br; 3 Departamento de Fisiologia e Biofísica, Instituto de Ciências Biomédicas I, Universidade de São Paulo, São Paulo 05508-000, Brazil; croc@icb.usp.br; 4 Departamento de Educação Física, Universidade Federal de Sergipe, São Cristóvão 49100-000, Brazil; fjaidar@gmail.com

**Keywords:** bodybuilders, physical activity, hypertrophy, strength, body size, anabolic-androgenic steroids, side effects, self-concept

## Abstract

Bodybuilding, as a high-performance sport, requires regular strength and resistance exercises with the principal objective of increasing muscle hypertrophy. However, many bodybuilders resort to the use of anabolic-androgenic steroids (AASs) to improve their performance in a short period of time. This study employs a survey-type, cross-sectional, descriptive–analytical method to evaluate the profile of bodybuilding athletes in the State of Sergipe, Brazil, and verify the level of knowledge/awareness about the health risks and impacts resulting from the use of such substances. Finite- and convenience-type populations are assessed, including individuals of both sexes, aged older than 18 years, self-declared bodybuilding athletes residing in the State of Sergipe, Brazil, and participating in regional and/or state competitions. As a result, no significant relationships were determined between sex (*p* = 0.492), age (*p* = 0.460), family income (*p* = 0.141), and medical follow-up sessions. For the variables level of education and medical follow-up vs. no follow-up sessions, a significant result was achieved (*p* = 0.01), with 74.3% of individuals reporting having follow-up treatment and 25.7% responding that they had no follow-up treatment, a percentage representing the group that completed their higher education. The substances most used by the athletes were Sustanon 250 or Durateston, Nandrolone Decanoate (Deca or Deca-Durabolin), and Testosterone. The most-reported acute side effects were acne at 33.8% (*n* = 20), irritability at 32.1% (*n* = 19), alopecia (hair loss), and nervousness at 23.7% (*n* = 14). The most-reported chronic side effects were arterial hypertension at 36.0% (*n* = 9), liver disease at 28.0% (*n* = 7), and cancer (non-specific) at 8.0% (*n* = 2). We concluded that, regardless of the athletes’ socioeconomic profiles, the use of AASs was high, with two or more substances being used in combination and for a prolonged period. Thus, it is necessary to promote awareness campaigns regarding the use of AASs and their effects on high-performance and recreational athletes.

## 1. Introduction

Bodybuilding is a high-performance sport that advocates the constant practice of strength and resistance exercises using the strength-training principle of progressive overload, with the main aim of promoting the hypertrophy of a set of muscle groups [1]. This muscular/strength development is acquired through the practice of continuous exercises that involve the movement of parts of the body, performed with free weights and/or machines designed for the target muscles [2,3,4].

In general, bodybuilding practitioners seek the best definition of muscle groups, which results in body sculpting and improvement in muscle volume and relief, with the goal of competing in national and/or international competitions [5,6]. The competitive sport requires the regular practice of specific training routines, in addition to the support of a multidisciplinary team composed of physical education professionals, nutritionists, physiotherapists, and endocrinologists [7,8]. Bodybuilding categories for males, as established by the International Federation of Bodybuilders (IFBB), are as follows: men’s bodybuilding, classic physique, and men’s physique. Female bodybuilding categories are bikini, wellness, body fitness, and women’s physique, which were created as a result of the increase in women’s attendance at gyms [9].

Bodybuilders consume a balanced diet and regularly train their muscles to guarantee increase in muscle mass. Some bodybuilders resort to the use of anabolic steroids and other substances that can increase muscle mass, which is associated with decrease in body fat percentage and increase in muscle strength, and improve the athletes’ performance over a shorter period of time [10]. The prolonged and unguided use of AASs can have deleterious multi-systemic effects on the body, including fertility problems, impotence and high blood pressure and cholesterol levels, as well as heart and liver anomalies, neuropsychiatric disorders, and dermatological problems [11,12,13,14]. However, when used in physiological dosages legally prescribed by a qualified doctor, they are generally safe and can also be helpful in chronic catabolic states, such as sarcopenia and hypoproliferative anemia [15,16]. The excessive use of anabolic steroids by males can result in infertility, testicular atrophy, testicular sensitivity, ejaculation problems, and erectile dysfunction [17]. In females, the development of gynecomastia, masculinization, anatomical changes in the vocal folds, menstrual cycle irregularity, and clitoral growth can occur [18,19].

Recreational and/or non-competitive bodybuilders report increased body satisfaction when using AASs, especially increases in muscle mass, strength, energy, speed, and recovery after performing intense exercise, which is associated with a lower body fat content [20]. Thus, the ambition to build the “ideal body” appears to be related to increased self-esteem and physical improvements promoted by the practice of strength exercises and the use of AASs [21]. The ideal body image projected and demanded by bodybuilding competitions has severe bio-psychological effects on the competitors, thus encouraging the use of AASs [21]. AASs affect an individual’s mood and can result in and enhance one’s emotional instability, depression, nervousness, violence, and aggressive behavior [17]. Depression, anxiety, behavioral changes, and AAS dependence are reported more frequently than physical problems [17,21].

The IFBB code of ethics is strict regarding the use of anabolic steroids and prohibits the use of these substances for the physical development of athletes who aim to increase their muscle mass and reduce fat content [9]. Some bodybuilders resort to the use of supplements and/or anabolic steroids to increase their strength and muscle mass without proper medical guidance, which can have harmful consequences on their health. Brazil is the second largest country regarding the number of bodybuilding competitors and fans [9], only behind the USA. In this sense, these professionals may be exposed to the risk of using AAS without guidance from a qualified health professional. From this perspective, this study was carried out to understand the socioeconomic–cultural profile and health status of bodybuilders in the State of Sergipe, since it allows a comprehensive understanding of the patterns of use of these substances in the region, including socioeconomic, cultural and behavioral aspects that can contribute to new decision-making and encourage the development of awareness campaigns by the Public Health System.

This study hypothesizes that the use of anabolic steroids without medical guidance among bodybuilders, as well as their motivations and expectations, in the state of Sergipe (in northeastern Brazil) are under-reported in the literature.

## 2. Materials and Methods

### 2.1. Sample and Study Design

This was a cross-sectional, descriptive–analytical, survey-type study, and the study population was finite and convenient. We adopted the following inclusion criteria: people of both sexes, aged older than 18 years, self-declared bodybuilding athletes, residents of any city in the state of Sergipe (in northeastern Brazil), and participants in regional and/or state competitions. The sample exclusion criteria included individuals younger than 18 years old; individuals who could not be contacted using virtual means; individuals with physical or mental illnesses that limited their understanding of questions or answers; and those who did not participate in regional and/or state competitions.

The research used a non-probabilistic accessibility sampling method to carry out a study on athletes who were registered with the Bodybuilding Federation of the state of Sergipe (Aracaju, Brazil). The number of responders was 104 participants out of 210 athletes (response rate = 50%) who were invited to participate in the study. Finally, after excluding incomplete responses and those lacking precise information, the final sample included 71 participants for data analysis: 60 men (84.5%), 10 women (14.1%) and 1 who preferred not to inform (1.4%). Following the administration of the questionnaire and collecting the data related to the use of anabolic steroids by bodybuilders in the state of Sergipe, the data were divided into two groups: namely, MG (male athletes) and FG (female athletes).

This study followed the Guidelines and Regulatory Norms for Research Involving Human Beings, present in Resolution 466/2012 of the National Health Council (https://conselho.saude.gov.br/resolucoes/2012/Reso466.pdf; accessed on 31 March 2024). All procedures performed during this study were approved by the Ethics Committee and research involving human beings of the Universidade Federal de Sergipe, according to number 5.635.795, approved on 12 September 2022.

### 2.2. Data Collection

The socioeconomic–cultural questionnaire and questions regarding the bodybuilders’ health were systematized using the Epidata 3.1 software. Data analysis was performed using Jamovi 2.3.26.0 [22], G* Power 3.1, and Excel (version 2021). Initially, the questionnaire contained 48 questions that were subdivided into three sub-areas: socioeconomic–cultural aspects, relationship with anabolic steroid use, and knowledge regarding side effects. We selected 22 questions and analyzed the answers using Jamovi 2.3.26.0 [22], G* Power 3.1, and Excel (version 2021).

The questionnaire was either sent to the participants by e-mail or directly administered to the participants whenever it was possible. The sample population was informed that all answers would remain confidential and that there was no obligation to answer the questions. The athletes’ information, such as e-mail address and telephone number for future contact, was kindly provided by the president of the Sergipe Federation of Bodybuilding (FESEMBB), Sergipe, Brazil.

### 2.3. Data Collection Procedure

The questionnaire was applied after its approval by the Research Ethics Committee of the Federal University of Sergipe—UFS (CAAE 59195822.3.0000.5546), thus complying with the Guidelines and Regulatory Norms for Research Involving Human Beings, present in the Resolution 466/2012 of the National Health Council—CNS. All participants were informed of the research procedures and agreed to sign the Free and Informed Consent Form (TCLE). The practitioners were informed that, at any time, they could choose to not answer and/or stop answering any question in the questionnaire. They were also informed regarding the guarantee of anonymity for each respondent. The questionnaire included questions on the participants’ sociodemographic and clinical information and their knowledge regarding the effects of using anabolic steroids.

## 3. Statistical Analysis

We obtained information on the participants’ gender, age, uses/used AAS education, ethnicity, income, and professional sector through univariate analysis of the data. Measures of central tendency, mean and dispersion, variance, and standard deviation values were obtained from the statistical analysis of the descriptive data. The descriptive data were evaluated using the Jamovi statistical program, version 2.3.26.0 for Windows. The dependent variable (i.e., the use of AASs) was related to the other study variables (monthly family income, medical consultations with the respective annual frequencies, period of time as an athlete, duration of AAS use, and number of AASs used simultaneously), in order to determine associations among the variables.

A chi-squared analysis (χ^2^) and Fisher’s exact test were used, when appropriate, with a *p*-value of *p* < 0.05 set as the significance threshold. Furthermore, the Phi and Cramer’s V tests were performed to verify the effect size of the associations among the variables. A probability of less than 5% was adopted to reject the null hypothesis or the lack of an association. The statistical analysis was performed using the Jamovi statistical program, version 2.3.26.0 for Windows.

## 4. Results

The data presented in Table 1 show the socioeconomic–cultural profiles of the bodybuilders in the State of Sergipe. The majority of the individuals, 84.5% (*n* = 60), were male; 14.1% (*n* = 10) were female; and 1.4% (*n* = 1) preferred not to identify their gender. Regarding age, around 46.4% (*n* = 32) were aged between 31 and 40 years, 43.5% (*n* = 30) were between 20 and 30 years, and 1.4% (*n* = 1) were older than 50 years. A total of 50.7% (*n* = 36) had completed higher education, 31% (*n* = 22) had not completed higher education, and 14.1% (*n* = 10) had completed high school. Only 1.4% (*n* = 1) had completed neither high school nor primary education (Table 1).

A total of 33.8% (*n* = 24) identified as white, 32.4% (*n* = 23) as black, 12.7% (*n* = 9) as brown, 11.3% (*n* = 8) as Asian, 2.8% (*n* = 2) as indigenous, 1.4% (*n* = 1) as mulatto, 1.4% (*n* = 1) as dark-skinned, and 2.8 (*n* = 2) did not declare color/race (Table 1). The income of the sample population was categorized as follows: no income, <minimum wage, 1–3 times the minimum wage, 3–6 times minimum wage, 6–9 times the minimum wage, and 9–12 times the minimum wage. We observed that 28.2% (*n* = 20) received between 1 to 3 and 3 to 6 times the minimum wage, 16.9% (*n* = 12) in the range of 6–9 times the minimum wage, 8.5% (*n* = 6) in the range of 9–12 times the minimum wage, 5.6% (*n* = 4) in the range of 12–15 times the minimum wage; 1.4% (*n* = 7) greater than >15 times the minimum wage, and 1.4% (*n* = 1) less than the minimum wage or no income (Table 1).

A total of 33.8% (*n* = 24) identified as freelance professionals and higher education teachers/technicians; 22.5% (*n* = 16) worked in commerce, banking, transport, hotels, or others; 11.3% (*n* = 8) worked as an employee of the federal, state, or municipal government; 8.5% (*n* = 6) considered themselves entrepreneurs; 4.2% (*n* = 3) worked outside the home, performing informal activities; 4.2% (*n* = 3) worked as personal trainers; and 2.8% (*n* = 2) worked in the industrial sector. Individuals who were self-employed, information producers, hospital receptionists, servers in mobile emergency care, security guards, Muay Thai teachers, or who did not work each presented a value of 1.4% (*n* = 1; Table 1).

### 4.1. Duration and Context of Anabolic Steroid Use 

A descriptive analysis was performed to detail the reason(s) for anabolic steroid use by the participants, and the following parameters were evaluated: duration of use in years, reasons for use during the off-season and pre-contest preparatory phases, means of prescription, ways of acquiring anabolic steroids, routes of administration, and the number of anabolic steroids simultaneously used (Table 2).

A total of 47.1% (*n* = 33) and 15.7% (*n* = 11) reported that they had been using/used AASs for over 2 years and between 6 and 12 months, respectively (Table 2). A total of 74.3% (*n* = 52) of athletes used AASs during the preparatory phase of off-season training to gain muscle mass. Furthermore, during the pre-contest stage, 42.9% (*n* = 30) of the athletes reported that they use/used AASs because they believed it burnt fat during this period (Table 2). Furthermore, the athletes were asked who suggested the use of AASs to them. The results show that 44.3% (*n* = 31) of the participants reported their coaches as the main source; physicians occupied the third position, at 11.4% (*n* = 8); and friends were the second most reported source, at 15.8% (*n* = 11; Table 2). A total of 36.8% (*n* = 25) reported that they obtained anabolic steroids from the internet and third parties. A total of 17.6% (*n* = 12) preferred not to respond to the question (Table 2).

The simultaneous use of two or three anabolic steroids was the most common answer, at 40.3% (*n* = 27) and 31.3% (*n* = 21), respectively. A total of 10.4% (*n* = 7) reported using only one substance, 7.5% (*n* = 5) reported simultaneously using four, and 3% (*n* = 2) simultaneously used five substances (Table 2). Injectable steroids were used the most, by 64.7% (*n* = 44) of the participants, 26.5% (*n* = 18) used anabolic steroids by two routes (injectable and oral), and 1.4% (*n* = 1) used the substances as a gel on the skin and orally (Table 2).

### 4.2. Main Steroids Used by Bodybuilding Athletes

Bodybuilding athletes use several substances to increase their lean body mass, improve their athletic and esthetic qualities, and burn fat. This practice is common among bodybuilding athletes and those who use it recreationally, that is, those who are not competitive athletes.

In a preliminary initial exploration of the main products used, as well as through interviews with some athletes to evaluate the initial scenario, athletes belonging to the FESEMBB under study listed some possible substances that they could (or could not) use (Table 3). Furthermore, considering the possibility of using substances not listed in the questionnaire, the item “Others” was included so that respondents could indicate AASs not included in the list.

In this context, we noticed that the interviewees simultaneously use/used more than one substance at different stages of competition (Table 3).

A total of 5.6% (*n* = 4) of the 71 participants reported that they do not currently use/did not use AASs, and 94.4% (*n* = 67) of the participants reported using AASs at some point during their time as athletes (Table 3). The main product used was Sustanon 250 or Durateston, at 28.98% (*n* = 20); Nandrolone decanoate (Deca or Deca-Durabolin), at 26.08% (*n* = 18); Testosterone enanthate and Trenbolone, at 23.18% (*n* = 16); Drostanolone Propionate and Stanozolol, at 21.73% (*n* = 15); and Boldenone, at 20.28% (*n* = 14; Table 3). The anabolic steroids with percentages lower than 15% were Testosterone cypionate (Deposteron), Metenolone enanthate (Primo or Primobolan), Methandrostenolone (Dianabol or D-bol), Oxandrolone (Anavar), and Oxymetholone (Anadrol or Hemogenin) (Table 3).

### 4.3. Sociodemographic Profiles of the Athletes and Their Adherence to Medical Monitoring

A correlation was established between the sociodemographic profiles and medical monitoring of the athletes who reported using/having used AASs (Table 4), which was determined using Fisher’s exact test for all variables. We also performed Phi and Cramer’s V tests to verify the effect size of the associations among the variables (Jamovi statistical program, version 2.3). There was no significant association between medical follow-up and the variables of gender (*p* = 0.492), age (*p* = 0.460), and family income (*p* = 0.141). The vast majority who completed higher education were supervised by a doctor, and vice versa (Table 4). 

On the other hand, there was a statistically significant association between athletes with a higher education who reported receiving or not receiving medical support: 74.3% (*n* = 26) received medical support, and 25.7% (*n* = 9) use/used AASs without a medical prescription (*p* = 0.01; Cramer’s V of 0.44, moderate relationship).

The level of association was weak (>0.1 to 0.29) (indicating that although the analysis indicated a statistically significant result, the associations were weak), moderate (0.3–0.49) (indicating that the variables were significantly associated, but with moderate strength), or strong (>0.5) (suggesting that data were strongly associated) (Table 4).

The correlation between ethnicity and medical support among those who reported using/have used AAS was evaluated (Table 4). For variable ethnicity, there was no statistically significant association for those who reported being white and receiving medical support; 63.0% (*n* = 15) reported using/have used AAS under medical supervision. However, in this population, 40.0% (*n* = 10) reported using/have used AAS without medical supervision. For those who reported being black, 50.0% (*n* = 11) who reported using/have used AAS reported having medical follow-up. However, 50.0% (*n* = 11) of this population reported using/have used AAS without medical supervision.

For those who reported being brown, 78.0% (*n* = 7) reported using/have used AAS under medical supervision, and 22.0% (*n* = 2) reported using/have used AAS without any medical supervision. For those who self-declared Asian and who reported using/having used AAS, 38.0% (*n* = 3) of this population received medical supervision, and 63.0% (*n* = 5) did not receive medical supervision (*p* = 0.446) (Table 4). 

The relationship between the occupation of bodybuilders who reported using/having used AAS and whether they received medical support was assessed. By occupation, freelance professionals accounted for the highest percentage of participants. Of this total, 78.0% (*n* = 18) reported having medical follow-up, and 22.0% (*n* = 5) did not have medical follow-up. 

Of those accounting for the second highest share by occupation (commerce, banking, transport, hotels or other services), 50.0% (*n* = 8) reported that they received medical follow-up, and 50.0% (*n* = 8) did not receive medical follow-up. Furthermore, for public employees (federal, state or municipal sphere), 50.0% (*n* = 4) reported receiving medical follow-up, and 50.0% (*n* = 4) did not receive medical follow-up. For professionals who reported being personal trainers, 67.0% (*n* = 2) reported having medical support, and 33.0% (*n* = 1) did not receive medical follow-up. Similar results were obtained for those who were not employed on the day of the interview, with 67.0% (*n* = 2) having medical follow-up and 33.0% (*n* = 1) not having it (*p* = 0.209) (Table 4).

### 4.4. Profiles of Anabolic Steroid Users and Their Knowledge Regarding AAS Side Effects

We conducted an analysis to assess the profiles of athletes who reported using/having used AASs compared to those of athletes who did not use or had not used AASs regarding the variables of gender, age, education, family income, and medical follow-up (Table 5).

A total of 20.3% (*n* = 12) of male athletes who reported using/having used AASs believed that these substances would not harm their health (Table 5). A total of 74.6% (*n* = 44; *p* = 0.177) used/had used AASs and believed that these substances harmed their health; there was no statistically significant result for the observed variables (Table 5). A total of 75.9% (*n* = 22) of male athletes aged between 20 and 30 years believed that the use of AASs would harm their health, while 20.7% (*n* = 6) believed that the use of AASs would not harm them. For athletes aged between 31 and 40 years, 63.6% (*n* = 21) responded that the use of AASs would harm their health, while 30.3% (*n* = 10) reported that it would not harm them.

A total of 75% (*n* = 27) of athletes who completed higher education believed that the use of AASs would harm their health, while 19.4% (*n* = 7) believed that the use of AASs would not harm them. A total of 65% (*n* = 13) of male athletes with incomplete higher education who reported using/having used AASs believed that the use of AASs would harm their health, while 30% (*n* = 6; *p* = 0.388) believed that the use of AASs would not harm their health (Table 5).

For the variable of ethnicity, of the total number of athletes who reported being white, 70.0% (*n* = 16) believed that the use of AASs could harm their health, and 25.0% (*n* = 6) did not believe that the use of AASs could harm their health (Table 5). For the total number of athletes who reported being black, 68.0% (*n* = 15) believed that the use f AASs could cause harm to their health, and 27.0% (*n* = 6) did not believe that AASs could cause harm to their health. For those who self-declared Asian, 67.0% (*n* = 6) believed that the use of AASs could harm their health, and 33.0% (*n* = 3) did not believe it. These results are similar to those of participants who reported being brown; 67.0% (*n* = 6) of this total believed that the use of AASs could harm their health, and 33.0% (*n* = 3) did not believe that AASs could harm their health (Table 5).

As for athletes who reported using/having used AASs, when stratified by the monthly family income criterion (Table 5), 63.2% (*n* = 12) of those earning 1–3 times the minimum wage believed that the use of AASs would cause harm to their health, and 26.3% (*n* = 5) believed that AASs would not cause harm to their health. As for athletes who reported using/having used AASs and who had family income of 3–6 times the minimum wage, 57.9% (*n* = 11) believed that the use of AASs would cause harm to their health, while 36.8% (*n* = 7) believed that the use of AASs would not cause harm to their health. For those reporting monthly family income of 6–9 times the minimum wage, 83.3% (*n* = 9) believed that the use of AASs would cause harm to their health), and 16.7% (*n* = 2) believed that AASs would not cause harm to their health. For those with family income of 12–15 times the minimum wage, 100% (*n* = 4) believed that the use of AASs would cause harm to their health), whereas 0.0% (*n* = 0) believed that AASs would not cause harm to their health. And, for those with family income above 15 times the minimum wage, 85.7% (*n* = 6) believed the use of AASs would cause harm to their health, and 14.3% (*n* = 1) believed that AASs would not cause harm to their health.

When evaluating the variable of occupation and whether or not the use of AASs causes harm to health (Table 5), among self-employed professionals, 75.0% (*n* = 18) of this population recognized that the use of AASs could cause harm to their health, and 21.0% (*n* = 5) did not believe that the use of AASs could cause harm to their health. For the second highest occupational prevalence reported (commerce, banking, transport, hotels or other services), 53.0% (*n* = 8) of this population recognized that the use of AASs could cause harm to their health, and 33.0% (*n*= 5) did not believe that the use of AASs could cause harm to their health. For those who reported being entrepreneurs, 100.0% (*n* = 5) recognized that the use of AASs could cause harm to their health. For public employees (federal, state or municipal sphere), 57.0% (*n* = 4) of this total believed that the use of AASs could cause harm to their health. In turn, 29.0% (*n* = 2) of this population believed that the use of AASs did not cause harm to their health (Table 5).

As for athletes who reported using/having used AASs, when stratified by the criterion of not having medical follow-up (Table 5), 73.3% (*n* = 22) believed the use of AASs would cause harm to their health), while 20.0% (*n* = 6) believed that AASs would not cause harm to their health. For those who reported receiving medical follow-up, 71.1% (*n* = 27) believed the use of AASs would cause harm to their health, while 26.3% (*n* = 10) believed that AASs would not cause harm to their health.

A descriptive analysis was performed to assess the main side effects reported by athletes who used/had used AASs as a way of preparing for the bodybuilding championships. The most significant acute side effects included acne, at 33.8% (*n* = 20); irritability, at 32.1% (*n* = 19); alopecia (hair loss), at 23.7% (*n* = 14); nervousness, at 23.7% (*n* = 14); irregular sleeping pattern, at 20.3% (*n* = 12); gynecomastia, at 15.2% (*n* = 9); and, finally, decreased spermatogenesis (difficulty in having children), at 11.9% (*n* = 7) (Table 6).

Furthermore, 16.7% (*n* = 10) reported not having experienced any side effects. In terms of the chronic side effects of AAS use by athletes, 36.0% (*n* = 9) reported high blood pressure levels, 28.0% (*n* = 7) liver disease, 8.0% (*n* = 2) cancer (non-specific), 4.0% (*n* = 1) heart problems, 4.0% (*n* = 1) insomnia, and 4.0% (*n* = 1) renal pathology. A total of 16.0% (*n* = 4) reported not having experienced any chronic side effect (Table 6).

## 5. Discussion

This study shows that the majority of athletes reported using and/or having used anabolic-androgenic steroids (AASs), with the majority of participants being aged between 31 and 40 years and with a use duration of above 2 years. AASs are mainly used to increase muscle mass and improve physical appearance. Bodybuilding athletes believe that achieving these goals is directly related to higher scores and ranking in the different categories of bodybuilding competitions [23]. 

Recent studies suggest that elite athletes, as well as recreational athletes who exercise in gyms, use these substances to improve their performance and/or appearance. In a study conducted on weightlifters, it was shown that AASs were used to gain muscle for competitive or personal reasons [21,23], corroborating results of our study, where a higher prevalence of AAS use was during the off-season phase (to gain muscle mass) and in the pre-contest phase (to accelerate fat burning), which is directly related to the aesthetic appearance for these athletes. In another study, 20% of professional athletes used AASs. This prevalence was higher among individuals from the USA, Brazil, Australia, and Nordic countries [24]. On the other hand, AAS use was lower in China, Korea, and Japan, suggesting that body image is not a priority in these countries [24]. 

The use of anabolic steroids (AASs) indiscriminately and without proper medical guidance can harm individuals and cause acne, increased hair growth, intense and progressive hair loss, stretch marks, blisters on the skin, contact dermatitis, rashes, and itching. Furthermore, a study has shown that, when used chronically, AASs can promote the development of liver cancer, which can lead to death [25,26]. 

However, the use of AASs and their chronic effects as cancer inducers in bodybuilders have not been fully elucidated in this study. Therefore, future studies are necessary to evaluate the duration of use, type of AAS used, and incidence of this disease in the population under study.

During the research, the most prevalent acute side effects were acne and alopecia (hair loss). Additionally, the chronic side effects were arterial hypertension, liver pathology and cancer (non-specific). Furthermore, behavioral changes related to well-being and mood were identified in this study among bodybuilders who used AASs, with irritability being the second most reported symptom among bodybuilders who used/had used AASs. Furthermore, the term “nervousness” was the fourth most prevalent acute side effect among respondents who used/had used AASs. 

### 5.1. The Association of the Use of AASs with Cardiovascular and Myocardial Dysfunction

In our study, the majority of athletes reported using/having used AASs for more than 2 years. The use involved the administration of two or more substances, with higher prevalence of associated AAS use.

According to the literature, the long-term use of AASs contributes to the development of myocardial dysfunction and accelerated coronary atherosclerosis [12,24,27,28,29]. During the functioning of the circulatory system, hemodynamic and biochemical events occur that ensure the optimized supply of oxygen and nutrients to peripheral tissues. Furthermore, the heart, as a central cardiac pump, triggers the systolic ejection of oxygen-rich blood into the arteries, leading to the perfusion of organs and tissues to maintain life, whether in physiological or altered conditions, such as during physical exercise, diabetes, and obesity [30,31,32,33]. Furthermore, hemodynamic homeostasis involves the precise regulation of blood pressure, coordinated by the autonomic nervous system and renal mechanisms, while blood composition is maintained by the processes of hematopoiesis and hormonal control [33,34,35,36]. 

However, it is important to highlight that the heart is one of the organs most frequently affected by the administration of anabolic steroids [37]. Direct myocardial injury caused by AASs promotes the marked hypertrophy of myocardial cells, extensive regional fibrosis, and necrosis. Unlike physiological hypertrophy, pathological hypertrophy decreases the strength of the heart [32,33,36]. High doses of AASs cause a significant increase in the concentration of erythrocytes and hemoglobin, which can lead to thromboembolism, intracardiac thrombosis, and stroke. Furthermore, the long-term use of AASs may contribute to a higher incidence of arrhythmia, atherosclerosis, the concentric myocardial hypertrophy of the left ventricle with impaired diastolic function, and sudden cardiac death [27,29,38,39].

### 5.2. The Association of AASs with the Circulatory System and Sexual Dysfunction

A systematic review and meta-analysis reported that the excessive and long-term use of AASs results in significant sperm changes, reduced testicular volume, and erectile dysfunction [40,41,42]. These changes suggest that the use of AASs negatively impacts male reproductive health. These changes were not mentioned by athletes, which does not mean that they will not develop these disorders if continued use occurs. What we emphasize is that adequate medical guidance is of fundamental importance to assess each patient and the real need for the use of these substances. In addition, an association of AAS abuse was found with gynecomastia and the prostate gland. Some studies describe a direct correlation between the indiscriminate use of AASs and the increased risk of developing gynecomastia, a phenomenon that appears to be a point of concern for those who reported using/having used AASs [43,44]. However, in our study, it was not the most prevalent acute side effect after AAS administration.

The main etiology of this phenomenon is associated with the ability of AASs to increase the concentration of circulating estrogen. This is due, in part, to the intensification of the aromatization of testosterone into estradiol after the use of these substances. As a consequence of this hormonal imbalance, the growth of breast tissue is evident among bodybuilders [42,43,44,45]. 

Surgery is not always indicated in gynecomastia associated with AASs, as the prevalence of meticulous intraoperative hemostasis and a careful glandular excision are common to minimize recurrence and achieve low complication rates [45]. Studies conducted on the effects of AAS use and abuse on the prostate gland have shown that the chronic use of these substances can promote prostatic hyperplasia and hypertrophy [42,46,47]. 

Prostatic hyperplasia is characterized by increase in the number of cells in the prostate, whereas hypertrophy refers to the increase in the size of cells in this endocrine environment. Furthermore, there is evidence that the prolonged use of AASs increases the risk of developing prostate cancer [42,46,47]. However, these authors also suggest that further studies are needed to corroborate this evidence.

The changes attributed to excessive and/or prolonged AAS use among females include menstrual irregularities (menarche, oligomenorrhea, secondary amenorrhea, and dysmenorrhea), anovulation, clitoral hypertrophy, changes in libido, and uterine atrophy [14,48,49]. Furthermore, many of these imbalances have permanent effects [42]. Furthermore, regardless of gender, some authors suggest that the use of AASs can trigger emotional disorders, such as those associated with impulsivity and emotional instability, as well as “borderline” personality disorder (rapid mood changes) [28,29,50].

### 5.3. Bodybuilders’ Knowledge of the Side Effects of Using AASs and How They Obtain Them

The most common means of obtaining AASs, as reported by the participants in our study, were “third party” and the internet. It is important to highlight that coaches and friends/users were the most common sources reported by the athletes. Physicians occupied the third position as professionals who prescribe AASs. Studies suggest that the desire to compete in bodybuilding competitions is the main motivation for participants to seek AASs, with study participants using a combination of oral and injectable medications. In our study, the highest prevalence of ASS administration was through the injectable route, corroborated by the substance (Sustanon 250 or Durateston) most reported by athletes (Table 3). Strength trainers and coaches were also identified as essential sources of information on AASs.

A very limited number of AAS users received information from healthcare professionals, and most of them experienced side effects but were willing to continue using AASs to achieve their desired physical appearance or to win competitions, which corroborates the results of Izzat (2023). It is important to highlight the low adherence to medical follow-up by AAS users. Concern regarding the means of purchasing low-quality products or those with unreported substances was highlighted, as the origin of products not prescribed by doctors is unknown, which can lead to the underreporting of the purchase of products of questionable quality from non-reputable companies without due compliance by government regulatory agencies [30,51].

This evidence suggests the need to develop awareness campaigns for this population, considering that AAS use without a proper medical prescription is prohibited.

The European Society of Endocrinology classifies AASs as substances capable of increasing the performance of professional and amateur athletes, in addition to being widely used to improve physical appearance. Furthermore, it highlights that anabolic steroid abuse can suppress the body’s ability to produce testosterone for several months [51].

According to the Food and Drug Administration (FDA), AASs are classified as type-III schedule-controlled substances, and their administration in the USA is restricted, which means that not only is a prescription required but that there are several additional controls, such that the prescription must be in writing and cannot be provided by a pharmacist [28]. Additionally, the labels on some steroid bottles recommend testing hormone levels while using these substances [28]. 

### 5.4. Steroid Use and Education

A recent study in the United Kingdom highlights that AAS users have lower levels of education compared to non-users. In our study, the highest prevalence of AAS use occurred in individuals with completed higher education. Furthermore, a statistically significant correlation was detected between athletes with higher education and those who reported having medical follow-up.

The majority of teenagers from the United Kingdom reported their first use of AASs at the age of 18 years. In addition, the study points out that 411,000 adults frequently used AASs [52]. Furthermore, young populations in other countries are also more likely to use AASs indiscriminately [23]. 

In medical practice, when faced with a patient whose health is potentially at risk after the chronic and/or frequent use of AASs, the main objective is to prevent, treat, and manage both the dependence on and consequences of long-term exposure to AAS use. The main factor that complicates both the diagnosis and care of this population is the clandestine and illegal use of these products by bodybuilding athletes and/or people who use these substances recreationally [53]. Clinicians and family doctors must be continuously educated regarding the adverse effects of AASs to investigate their use by high-risk patients, especially young athletes [12].

We must emphasize once again that the IFBB code of ethics is strict regarding the prohibition of the use of AASs with the aim of obtaining muscle mass and reducing body fat by bodybuilding athletes [9]. It is fully known that their use without guidance from a qualified doctor can contribute to the development of harmful effects on the body that can result in death [32,54]. In this sense, it is imperative to increase the number of campaigns that raise awareness regarding the adverse effects of the indiscriminate and unguided use of AASs.

However, the changes that occur due to the indiscriminate use of AASs are reversible in most cases. Thus, patients can be offered a good prognosis and should be advised regarding good compliance with management plans to maintain good health [29].

## 6. Conclusions

We can conclude that a considerable number of the studied athletes used/had used AASs, regardless of their socioeconomic profile. Furthermore, the simultaneous and long-term use of more than one of these substances through different administration routes (injectable and oral) was the most common behavior reported by the athletes.

The level of education does not reflect a reduced use of AASs by athletes. Furthermore, the groups that guided and/or motivated athletes to use AASs were friends and coaches, suggesting that it is necessary to raise awareness regarding the use of AASs and their harmful effects on bodybuilding athletes and the general population, as they can become a serious public health problem.

## Figures and Tables

**Table 1:** Socioeconomic–cultural profiles of the bodybuilding athletes in the State of Sergipe, Brazil.

Variable	*n* (Prevalence %)	Mean (SD)	*p*-Value
Gender			
Male	60 (84.5)	31.7 (6.2)	0.915
Female	10 (14.1)	33.7 (7.1)	
I prefer not to answer	1 (1.4)	-	
Age			
20–30 years	30 (43.5)	31.92 (6.32)	0.188
31–40 years	32 (46.4)		
41–50 years	6 (8.7)		
Over 50 years	1 (1.4)		
Do/did you use AASs?			
YES	67 (94.4)	n/a	n/a
NO	4 (5.6)		
Education			
Complete primary education	1 (1.4)	n/a	n/a
Incomplete high school	1 (1.4)		
Complete high school	10 (14.1)		
Incomplete higher education	22 (31.0)		
Complete higher education	36 (50.7)		
Ethnicity			
White	24 (33.8)	n/a	n/a
Asian	8 (11.3)		
Brown	9 (12.7)		
Black	23 (32.4)		
Indigenous	2 (2.8)		
Mulatto	1 (1.4)		
Dark-skinned	1 (1.4)		
Not declared	2 (2.8)		
Income			
No income	1 (1.4)	n/a	
<minimum wage (<CHF 253.53 per month)	1 (1.4)		
1–3 times minimum wage (CHF 253.53–760.59 per month)	20 (28.2)		
3–6 times minimum wage (CHF 760.59–1521.18 per month)	20 (28.2)		
6–9 times minimum wage (CHF 1521.18–2281.77 per month)	12 (16.9)		
9–12 times minimum wage (CHF 2281.77–3042.36 per month)	6 (8.5)		
12–15 times minimum wage (CHF 3042.36–3802.95 per month)	4 (5.6)		
>15 times minimum wage (>CHF 3802.95 per month)	7 (1.4)		
Occupation/profession			
Self-employed	1 (1.4)	n/a	n/a
Condominium administration	1 (1.4)		
Public employee (federal, state, or municipal)	8 (11.3)		
In commerce (banking, transport, hotels, etc.)	16 (22.5)		
Personal trainer	3 (4.2)		
Muay Thai teacher	1 (1.4)		
Freelance professional	24 (33.8)		
Hospital reception	1 (1.4)		
Mobile emergency service server	1 (1.4)		
Works outside the home in informal activities	3 (4.2)		
Security guard	1 (1.4)		
Entrepreneur	6 (8.5)		
Industry	2 (2.8)		
Does not work	3 (4.2)		

**Abbreviations:** AASs: anabolic-androgenic steroids; *n*: number of respondents; %: prevalence in percentages obtained from the descriptive statistics of the observed variables (frequency table); >: greater than; <: less than; Mean: average; SD: standard deviation; n/a: not applicable. *p*-values obtained for gender and age were evaluated using the Shapiro–Wilk test; other values for the group are expressed as the mean (SD). Jamovi statistical program (version 2.3) was retrieved from (https://www.jamovi.org accessed on 17 April 2023). In Brazil, the minimum wage corresponds to BLR 1412.00 per month worked (40 h per month worked) (https://www.planalto.gov.br/ccivil_03/_ato2023-2026/2023/decreto/D11864.htm—accessed on 23 March 2024). This value, converted to CHF, corresponds to CHF 253.43 per month worked (40 h per month worked) (https://www.xe.com/pt/currencyconverter/; accessed on 23 March 2024).

**Table 2:** Context and duration of anabolic steroid use.

Variable	*n* (Prevalence %)
Usage time	
Between 0 and 3 months	6 (8.6)
Between 3 and 6 months	7 (10.0)
Between 6 and 12 months	11 (15.7)
Between 12 and 24 months	10 (14.3)
Over 2 years	33 (47.1)
Does not use/did not use	3 (4.3)
Reason for use during the off-season	
Muscle mass gain	52 (74.3)
Greater training volume	7 (10.0)
Post-workout recovery	5 (7.1)
Clinical pathology treatment	2 (2.9)
Does not use/did not use	4 (5.7)
Reason for use during the pre-contest phase	
Improve training intensity	19 (27.1)
Burn fat	30 (42.9)
Post-workout recovery	15 (21.4)
Clinical pathology treatment	1 (1.4)
Does not use/did not use	5 (7.1)
Means of prescription	
Friends	11 (15.8)
Coach	31 (44.3)
Gym acquaintances	4 (5.7)
Internet	3 (4.3)
Nutritionist	4 (5.7)
Physician	8 (11.4)
Physical education professional	3 (4.3)
Studying (own expense)	1 (1.4)
Several of the above	1 (1.4)
Laboratory sponsorship	1 (1.4)
Does not use/did not use	3 (4.3)
Means of acquiring anabolic steroids	
Internet	25 (36.8)
Third party	25 (36.8)
Pharmacy	4 (5.9)
Prefer not to respond	12 (17.6)
Not applicable	2 (2.9)
Number of anabolic steroids simultaneously used	
Zero	5 (7.5)
One	7 (10.4)
Two	27 (40.3)
Three	21 (31.3)
Four	5 (7.5)
Five	2 (3.0)
Route of administration	
Injectable	44 (64.7)
Gel	1 (1.4)
Oral	1 (1.4)
Both	18 (26.5)
Does not use/did not use	4 (5.9)

*n*: number of respondents; %: prevalence in percentages. *n* and % were obtained from the descriptive statistics of the observed variables (frequency table). Jamovi statistical program (version 2.3) was retrieved from (https://www.jamovi.org, accessed on 17 April 2023).

**Table 3:** Main AASs currently being used by the interviewed athletes.

Variable	*n* (Prevalence %)
Products used/currently being used *	
Sustanon 250 or Durateston	20 (28.98)
Nandrolone decanoate (Deca or Deca-Durabolin)	18 (26.08)
Testosterone Enanthate	16 (23.18)
Trenbolone	16 (23.18)
Drostanolone Propionate	15 (21.73)
Stanozolol	15 (21.73)
Boldenone	14 (20.28)
Testosterone cypionate (Deposteron)	10 (14.49)
Metenolone enanthate (Primo or Primobolan)	9 (13.04)
Methandrostenolone (Dianabol or D-bol)	8 (11.59)
Oxandrolone (Anavar)	7 (10.14)
Oxymetholone (Anadrol or Hemogenin)	1 (1.44)

*n*: number of respondents; %: prevalence in percentages. *n* and % were obtained from the descriptive statistics of the observed variables (frequency table). Jamovi statistical program (version 2.3) was retrieved from (https://www.jamovi.org, accessed on 17 April 2023). * Note: The athletes were instructed to select more than one option, if applicable.

**Table 4:** Correlation between the sociodemographic profiles and medical monitoring of anabolic steroid users.

Variables	No Medical Follow-Up N (%)	Received Medical Follow-Up N (%)	*p*-Value	Cramer’s V Effect Size
Gender	30 (100)	40 (100)	0.492	0.15
Male	24 (40.7)	35 (59.3)		
Female	5 (50.0)	5 (50.0)		
Prefer not to answer	1 (100)	0 (0.0)		
Age	30 (100)	40 (100)	0.460	0.20
20 to 30 years	15 (50.0)	15 (50.0)		
31 to 40 years	12 (36.4)	21 (63.6)		
41 to 50 years	2 (33.3)	4 (66.7)		
>50 years	1 (100)	0 (0.0)		
Education	30 (100)	40 (100)	0.01	0.44
Complete primary education	1 (0.0)	0 (0.0)		
Incomplete high school	1 (100)	0 (0.0)		
Complete high school	6 (60.0)	4 (40.0)		
Incomplete higher education	13 (59.1)	9 (40.9)		
Complete higher education	9 (25.7)	26 (74.3)		
Postgraduate education	0 (0.0)	1 (100)		
Ethnicity	30 (100)	40 (100)	0.446	0.29
Asian	5 (63.0)	3 (38.0)		
White	10 (40.0)	15 (60.0)		
Brown	1 (100)	0 (0.0)		
Mulatto	0 (0.0)	1 (100)		
Black	11 (50.0)	11 (50.0)		
Indigenous	1 (50.0)	1 (50.0)		
Dark-skinned	2 (22.0)	7 (78.0)		
Not declared	0 (0.0)	2 (100)		
Monthly family income	30 (100)	40 (100)	0.141	0.29
<minimum wage (<CHF 253.53 per month)	0 (0.0)	1 (100)		
1–3 times minimum wage (CHF 253.53–760.59 per month)	8 (42.1)	11 (57.9)		
3–6 times minimum wage (CHF 760.59–1521.18 per month)	8 (40.0)	12 (60.0)		
6–9 times minimum wage (CHF 1521.18–2281.77 per month)	4 (33.3)	8 (66.7)		
9–12 times minimum wage (CHF 2281.77–3042.36 per month)	5 (83.3)	1 (16.7)		
12–15 times minimum wage (CHF 3042.36–3802.95 per month)	3 (75.0)	1 (25.0)		
>15 minimum wages (>CHF 3802.95 per month)	1 (100)	0 (0.0)		
Occupation/profession	30 (100)	40 (100)	0.209	0.26
Self-employed	0 (0.0)	1 (100)		
Public employee (federal, state, or municipal)	4 (50.0)	4 (50.0)		
In commerce (banking, transport, hotels, etc.)	8 (50.0)	8 (50.0)		
Personal trainer	1 (33.0)	2 (67.0)		
Muay Thai teacher	1 (100)	0 (0.0)		
Freelance professional	5 (22.0)	18 (78.0)		
Hospital reception	1 (100)	0 (0.0)		
Mobile emergency service server	1 (100)	0 (0.0)		
Security guard	0 (0.0)	1 (100)		
Entrepreneur	4 (80.0)	1 (20.0)		
Industry	1 (50.0)	1 (50.0)		
Condominium administration	1 (100)	1 (100)		
Works outside the home in informal activities	2 (50.0)	2 (50.0)		
Does not work	1 (33.0)	2 (67.0)		

**Abbreviations:** N: number of respondents; %: prevalence in percentages; >: greater than; <: less than; *p*-value: *p*-values obtained using Fisher’s exact test or χ^2^. The level of association according to Cramer’s V effect size is weak (>0.1 to 0.29), moderate (0.3–0.49), or strong (0.5). Jamovi statistical program (version 2.3) was retrieved from (https://www.jamovi.org. accessed on 17 April 2023).

**Table 5:** Correlation between athletes’ profiles regarding their knowledge of the side effects of AASs.

Variables	Do You Believe That the Use of AASs Will Harm Your Health?			*p*-Value	Cramer’s V Effect Size
	Does Not Use N (%)	No N (%)	Yes N (%)		
Gender	4 (100)	16 (100)	49 (100)	0.177	0.19
Male	3 (5.1)	12 (20.3)	44 (746)		
Female	1 (11.1)	3 (33.3)	5 (55.6)		
Prefer not to answer	0 (0.0)	1 (100)	0 (0.0)		
Age	4 (100)	16 (100)	49 (100)	0.484	0.18
20 to 30 years	1 (3.4)	6 (20.7)	22 (75.9)		
31 to 40 years	2 (6.1)	10 (30.3)	21 (63.6)		
41 to 50 years	1 (16.7)	0 (0.0)	5 (83.3)		
>50 years	0 (0.0)	0 (0.0)	1 (100)		
Education	4 (100)	30 (100)	40 (100)	0.388	0.26
Complete primary education	0 (0.0)	(0.0)	1 (100)		
Incomplete high school	0 (0.0)	1 (100)	0 (0.0)		
Complete high school	1 (10.0)	1 (10.0)	8 (80.0)		
Incomplete higher education	1 (5.0)	6 (30.0)	13 (65.0)		
Complete higher education	2 (5.6)	7 (19.4)	27 (75.0)		
Postgraduate education	0 (0.0)	0 (0.0)	1 (100)		
Ethnicity	4 (100)	16 (100)	49 (100)	0.990	0.25
Asian	1 (13.0)	1 (13.0)	6 (67.0)		
White	2 (8.0)	6 (25.0)	16 (70.0)		
Brown	0 (0.0)	0 (0.0)	1 (100)		
Mulatto	0 (0.0)	0 (0.0)	1 (100)		
Black	1 (5.0)	6 (27.0)	15 (68.0)		
Indigenous	0 (0.0)	0 (0.0)	2 (100)		
Dark-skinned	0 (0.0)	3 (33.0)	6 (67.0)		
Not declared	0 (0.0)	0 (0.0)	2 (100)		
Monthly family income	4 (100)	30 (100)	40 (100)	0.952	0.23
<minimum wage (<CHF 253.53 per month)	0 (0.0)	0 (0.0)	1 (100)		
1–3 times minimum wage (CHF 253.53–760.59 per month)	2 (10.5)	5 (26.3)	12 (63.2)		
3–6 times minimum wage (CHF 760.59–1521.18 per month)	1 (5.3)	7 (36.8)	11 (57.9)		
6–9 times minimum wage (CHF 1521.18–2281.77 per month)	1 (8.3)	2 (16.7)	9 (83.3)		
9–12 times minimum wage (CHF 2281.77–3042.36 per month)	0 (0.0)	1 (16.7)	5 (83.3)		
12–15 times minimum wage (CHF 3042.36–3802.95 per month)	0 (0.0)	0 (0.0)	4 (100)		
>15 times minimum wage (>CHF 3802.95 per month)	0 (0.0)	1 (14.3)	6 (85.7)		
Occupation/profession	4 (100)	16 (100)	49 (100)	0.898	0.19
Self-employed	0 (0.0)	0 (0.0)	1 (100)		
Public employee (federal, state, or municipal)	1 (14.0)	2 (29.0)	4 (57.0)		
In commerce (banking, transport, hotels, etc.)	2 (13.0)	5 (33.0)	8 (53.0)		
Personal trainer	0 (0.0)	0 (0.0)	3 (100)		
Muay Thai teacher	0 (0.0)	0 (0.0)	1 (100)		
Freelance professional	1 (4.0)	5 (21.0)	18 (75.0)		
Hospital reception	0 (0.0)	0 (0.0)	1 (100)		
Mobile emergency service server	0 (0.0)	0 (0.0)	1 (100)		
Security guard	0 (0.0)	0 (0.0)	1 (100)		
Entrepreneur	0 (0.0)	0 (0.0)	5 (100)		
Industry	0 (0.0)	1 (50.0)	1 (50.0)		
Condominium administration	0 (0.0)	1 (100)	0 (0.0)		
Works outside the home in informal activities	0 (0.0)	1 (25.0)	3 (75.0)		
Does not work	0 (0.0)	1 (33.0)	2 (67.0)		
Medical follow-up	3 (100)	16 (100)	49 (100)	0.667	0.12
No	2 (6.7)	6 (20.0)	22 (73.3)		
Yes	1 (2.6)	10 (26.3)	27 (71.1)		

**Abbreviations:** N: number of respondents; %: prevalence in percentages; *p*-value: *p*-values obtained using Fisher’s exact test or χ^2^. The level of association according to Cramer’s V effect size is weak (>0.1 to 0.29), moderate (0.3–0.49), or strong (0.5). Jamovi statistical program (version 2.3) was retrieved from (https://www.jamovi.org, accessed on 17 April 2023).

**Table 6:** Main acute and chronic side effects of AAS use reported by athletes.

**Acute Side Effects**	***n* (Prevalence %)**
Acne	20 (33.8)
Irritability	19 (32.1)
Alopecia (hair loss)	14 (23.7)
Nervousness	14 (23.7)
Sleep imbalance	12 (20.3)
Gynecomastia	9 (15.2)
Decreased spermatogenesis (difficulty having children)	7 (11.9)
Did not indicate/did not have any *	10 (16.7)
**Chronic Side Effects**	***n* (Prevalence %)**
Arterial hypertension	9 (36.0)
Liver pathology	7 (28.0)
Cancer (non-specific)	2 (8.0)
Cardiac problems	1 (4.0)
Insomnia	1 (4.0)
Kidney pathology	1 (4.0)
Did not indicate/did not have any *	4 (16.0)

**Abbreviations:** *n*: number of respondents; %: prevalence in percentages. * Note: A total of 11 out of the 69 participants did not respond to whether they experienced acute side effects, and 46 did not respond to whether they experienced chronic side effects. The participants who responded and indicated that they did not experience any side effect, either acute or chronic, were categorized as “Did not indicate/did not have any”: Athletes were instructed to select more than one option, if applicable. N and % were obtained from the descriptive statistics of the observed variables (frequency table). Jamovi statistical program (version 2.3) was retrieved from (https://www.jamovi.org, accessed on 17 April 2023).

## Data Availability

All original materials prepared for the study are included in the article, and further enquiries can be addressed to the corresponding author(s).
